# Применение метформина у больных сахарным диабетом 2 типа при остром инфаркте миокарда: безопасность и влияние на гликемический контроль

**DOI:** 10.14341/probl13170

**Published:** 2023-02-25

**Authors:** М. А. Коротина, И. Г. Починка, Л. Г. Стронгин

**Affiliations:** Приволжский исследовательский медицинский университет; Приволжский исследовательский медицинский университет; Приволжский исследовательский медицинский университет

**Keywords:** метформин, сахарный диабет 2 типа, острый инфаркт миокарда, гликемический контроль, острое повреждение почек

## Abstract

**ОБОСНОВАНИЕ:**

ОБОСНОВАНИЕ. Инфаркт миокарда (ИМ) у больных сахарным диабетом 2 типа (СД2) встречается в 1,5–3,0 раза чаще, чем в общей популяции. Метформин противопоказан больным СД2 при остром коронарном синдроме из-за риска развития лактат-ацидоза. Возможность применения метформина за пределами первых 48 ч ИМ остается актуальным вопросом, что может способствовать повышению безопасности пациентов.

**ЦЕЛЬ:**

ЦЕЛЬ. Оценить безопасность и качество гликемического контроля при применении метформина у больных СД2 во время стационарного этапа лечения острого ИМ

**МАТЕРИАЛЫ И МЕТОДЫ:**

МАТЕРИАЛЫ И МЕТОДЫ. В одноцентровое исследование включали больных СД2, последовательно госпитализированных с острым ИМ с подъемом сегмента ST и подвергнутых чрескожному коронарному вмешательству (ЧКВ), всего включен 161 пациент. Медиана времени инициации терапии метформином составила 5 сут от момента поступления. Уровень креатинина оценивался при поступлении и через 48 ч после ЧКВ. Кислотно-щелочное состояние крови (КЩС) и уровень лактата оценивались при поступлении и на 3-и сутки после начала приема метформина. Критерием эффективности гликемического контроля считали значение доли измерений гликемии в пределах целевого диапазона 6,1–10,0 ммоль/л во время госпитализации («hospital time in range», hTIR), критическим уровнем считали hTIR >55%. Отдаленный исход оценивался на 365-й день от момента госпитализации.

**РЕЗУЛЬТАТЫ:**

РЕЗУЛЬТАТЫ. В стационаре метформин был назначен 99 пациентам (61%; группа «М+»), 62 пациента составили группу «М-». Применение метформина сопровождалось более качественным гликемическим контролем в группе «М+» по сравнению с группой «М-»: среднее значение гликемии 9,3±1,6 vs 10,3±2,3 ммоль/л (р=0,002), SD гликемии 2,87±1,1 vs 3,26±1,8 (p=0,049), hTIR 60±18% vs 48±23% (p<0,001). В группе «М+» на 3-и сутки от начала использования метформина отмечаются достоверные, но клинически незначимые изменения КЩС, уровень лактата не увеличивался, случаев лактат-ацидоза в исследуемой когорте не выявлено. Применение метформина до госпитализации по поводу ИМ не сопровождалось увеличением риска развития острого повреждения почек (ОПП): ОР 0,85 (0,37–1,96), p=0,691.

**ЗАКЛЮЧЕНИЕ:**

ЗАКЛЮЧЕНИЕ. У больных СД2, госпитализированных по поводу ИМ, использование метформина ассоциировано с более качественным гликемическим контролем — более низкими уровнями средней гликемии, ее вариабельности, увеличением времени пребывания hTIR. Проведение ангиографии у больных, исходно получающих терапию метформином, не сопровождается повышением риска развития ОПП. Назначение метформина на 3–7-и сутки после ангиографии не приводит к повышению уровня лактата и клинически значимым отклонениям показателей КЩС.

## Abstract (en)

**BACKGROUND:**

BACKGROUND: Myocardial infarction (MI) in patients with diabetes type 2 (T2DM) occurs 1.5–3.0 times higher than in general population. Metformin is contraindicated for patients with T2DM and acute coronary syndrome due to the risk of developing lactic acidosis. Using metformin more than 48 hours of MI is a topical question, which will help to improve patient’s safety.

**AIM:**

AIM: To evaluate the safety and quality of glycemic control using metformin in patients with T2DM during inpatient treatment for MI

**MATERIALS AND METHODS:**

MATERIALS AND METHODS: The study included 161 patients with T2DM, who were hospitalized consecutively with acute MI with ST-elevation and underwent percutaneous coronary intervention (PCI). Average time of metformin initiation was 5th day from admission. Creatinine was assessed at admission and 48 hours after PCI. The acid-base balance and lactate were assessed at admission and on the 3rd day after the start of using metformin. Criteria for the effectiveness of glycemic control was the proportion of glycemic measurements in target range of 6.1–10.0 mmol/l during hospitalization (“hospital time in range”, hTIR). hTIR >55% was considered to be a critical level. The long-term outcome was estimated at 365 days after hospitalization.

**RESULTS:**

RESULTS: Metformin was prescribed to 99 patients (61%) (“M+”group) during the hospitalization, 62 patients were in “M-”group. Use of metformin was accompanied with better glycemic control in the «M+» group compared to the «M-»: mean glycemia 9.3 ± 1.6 vs 10.3 ± 2.3 mmol/l (p=0,002), SD 2.87 ± 1.1 vs 3.26 ± 1.8 (p=0,049), hTIR 60 ± 18% vs 48 ± 23% (p<0,001). There were clinically insignificant changes in acid-base balance on the 3rd day from the start of metformin use in the “M+” group, the lactate level did not increase. Use of metformin before to hospitalization with MI was not associated with an increased risk of developing acute kidney injury (AKI): RR 0.85 (0.37–1.96), p=0,691.

**CONCLUSION:**

CONCLUSION: Use of metformin in patients with T2DM and acute MI is associated with better glycemic control. Carrying out angiography in patients, treated with metformin before the hospitalization, is not accompanied by an increased risk of developing AKI. Appointment of metformin in 3–7 days after angiography does not lead to an increase level of lactate and significant deviations in acid-base balance.

## ОБОСНОВАНИЕ

Известно, что сахарный диабет 2-го типа (СД2) ассоциирован с повышенным сердечно-сосудистым риском. Острый инфаркт миокарда (ИМ) у больных СД2 встречается в 1,5–3,0 раза чаще, чем в общей популяции [1], не менее четверти всех больных острым ИМ страдают СД2 [2]. Несмотря на достижения последних двух десятилетий в лечении острого коронарного синдрома (ОКС) за счет внедрения эндоваскулярных коронарных вмешательств, летальность больных СД2 в 1,5–2,0 выше, чем лиц, не имеющих диабета [3].

По-прежнему актуальным остается вопрос применения различных групп сахароснижающих препаратов у больных СД2 при ИМ. Согласно текущим российским рекомендациям, ОКС не является обязательным показанием для перевода на инсулинотерапию, и предусматривается возможность применения оральных сахароснижающих препаратов [4]. В то же время применение некоторых лекарственных групп при ИМ ограничено: рекомендуется отмена бигуанидов и тиазолидиндионов; отмечается отсутствие данных о безопасности при ОКС ингибиторов натрий-глюкозного ко-транспортера 2 типа (иНГЛТ-2), агонистов глюкагоноподобного пептида-1 и ингибиторов дипептидилпептидазы 4 типа [4]. Указывается, что метформин противопоказан больным СД2 при ОКС из-за риска развития лактат-ацидоза на фоне тканевой гипоксии и неизученного влияния на ранние и отдаленные клинические исходы ОКС [4]. Дополнительным фактором, ограничивающим применение метформина при ИМ, является признание комбинации метформина с йодсодержащими рентгеноконтрастными веществами противопоказанной, в инструкции к препарату имеется указание на необходимость соблюдать 48-часовой интервал до назначения метформина после использования контраста.

В то же время в исследовании United Kingdom Prospective Diabetes Study (UKPDS) было показано, что применение метформина снижает риск смерти, связанной с диабетом, на 42%, смерти по любой причине — на 36%, а также острого ИМ — на 39% (р=0,01) [5]. Показательно, как после публикаций результатов крупных наблюдательных исследований и анализа баз данных реальной клинической практики менялось отношение к использованию метформина при хронической сердечной недостаточности — от указанного в инструкции к препарату противопоказания до признания наиболее безопасным препаратом [6–8]. Исследования последних лет раскрывают механизмы кардиопротективных эффектов метформина. Показано, что метформин ослабляет ремоделирование миокарда и воспалительную реакцию при экспериментальном инфаркте у крыс [9], применение препарата сопровождается снижением апоптоза кардиомиоцитов [10], уменьшением ишемического и реперфузионного повреждения у больных СД2 [11–13].

Имеющееся на сегодняшний день в инструкции противопоказание к использованию метформина при остром ИМ формально ограничивает его применение в течение 28 дней от момента появления симптомов, именно такая продолжительность ИМ предусмотрена четвертым универсальным определением ИМ [14]. С другой стороны, внедрение современных реперфузионных технологий позволило снизить частоту развития осложнений ИМ и существенно сократить пребывание пациента в стационаре. В большинстве случаев пациент в стабильном состоянии уже на 3–5-е сутки может завершить госпитализацию. Поэтому возможность применения метформина за пределами первых 48 ч ИМ остается актуальным вопросом, ответ на который позволит не только снять формальные противоречия, но и способствовать повышению безопасности пациентов.

## ЦЕЛЬ ИССЛЕДОВАНИЯ

Оценить безопасность и качество гликемического контроля при применении метформина у больных СД2 во время стационарного этапа лечения острого ИМ.

## МАТЕРИАЛЫ И МЕТОДЫ

Место и время проведения исследования

Место проведения. Региональный сосудистый центр ГБУЗ НО «Городская клиническая больница №13» города Нижнего Новгорода.

Время исследования. Включение больных в исследование проводилось в течение 8 мес 2021 г. Отдаленный исход оценивался на 365-й день от момента госпитализации.

Изучаемые популяции

Использовался сплошной способ формирования изучаемой популяции. Включались больные СД2, последовательно госпитализированные с острым ИМ с подъемом сегмента ST, подвергнутые чрескожному коронарному вмешательству (ЧКВ) и подписавшие информированное согласие. Диагнозы острый ИМ и СД2 устанавливались на основании текущих клинических рекомендаций [4][15].

Критерии исключения: смертельный исход в течение 1-х суток госпитализации.

Общее количество включенных в исследование пациентов составило 161.

Дизайн исследования

Одноцентровое проспективное интервенционное нерандомизированное исследование.

Описание медицинского вмешательства (для интервенционных исследований)

Управление гликемией и СД2 в стационаре осуществлялось следующим образом. При поступлении в стационар при наличии одного из следующих критериев: 1) применение инсулинотерапии до ИМ, 2) гликемия при поступлении ≥12,1 ммоль/л, 3) наличие острой сердечной недостаточности, 4) расчетная скорость клубочковой фильтрации (рСКФ) <30 ммоль/л пациенту назначалась монотерапия инсулинами [16], в дальнейшем с учетом динамики результатов лабораторных исследований рассматривался вопрос о назначении таблетированных сахароснижающих препаратов (ТСП). При отсутствии перечисленных выше критериев ТСП могли назначаться уже в 1-е сутки госпитализации за исключением метформина и иНГЛТ-2, решение о применении которых принималось не раньше чем через 48 ч после ангиографии.

Решение о возможности возобновления терапии метформином или необходимости его назначения впервые принималось с учетом отсутствия признаков острой сердечной и острой печеночной недостаточности, отсутствия кетонов в моче, нормального значения лактата и уровня рСКФ>45 мл/мин. Уровень креатинина оценивался при поступлении в стационар и через 48 ч после проведения ангиографии. Критериями острого повреждения почек (ОПП) считали повышение креатинина >44 мкмоль/л или >25% исходного значения. У больных с ОПП метформин мог быть назначен позднее в случае возвращения рСКФ к исходному уровню в динамике. Кислотно-щелочное состояние крови (КЩС) и уровень лактата оценивались при поступлении и на 3-и сутки после начала применения метформина. Гликемия при поступлении измерялась вне зависимости от последнего приема пищи, со 2-х суток гликемия исследовалась натощак и перед основными приемами пищи. Медиана продолжительности стационарного лечения составила 10 [ 8; 12] дней. Медиана количества измерений гликемии в течение госпитализации составила 21 [ 14; 29] раз. Качество гликемического контроля оценивали по средней гликемии, стандартному отклонению (SD), количеству случаев гипогликемии (уровень глюкозы <3,9 ммоль/л). Критерием эффективности гликемического контроля считали значение доли измерений гликемии в пределах целевого диапазона 6,1–10,0 ммоль/л во время госпитализации («hospital time in range», hTIR), критическим уровнем считали hTIR>55% (в предшествующих работах авторов было показано, что при таком значении hTIR во время стационарного лечения по поводу острого ИМ достоверно снижается риск наступления летального исхода в течение 1 года после госпитализации) [16].

Статистический анализ

Статистическая обработка проводилась в программе Statistica (StatSoft Inc. США, версия 10.0). Количественные данные представлены в виде среднего арифметического ± стандартное отклонение (Mean±SD), медиан и интерквартильных интервалов (Median [Q1; Q3]). Для оценки достоверности различий количественных данных использовался тест Манна–Уитни, долей — χ2 Пирсона.

Этическая экспертиза

Исследование было одобрено на заседании подкомиссии по рассмотрению диссертационных работ Комитета по этике ФГБОУ ВО «Приволжский медицинский исследовательский университет» Минздрава России, протокол №3/Д-2021 от 09.04.2021 года. Представляемая работа является фрагментом интервенционной части исследования.

## РЕЗУЛЬТАТЫ

До госпитализации метформин получали 89 больных, суточная доза составила 1500 [ 1000; 2000] мг. За время стационарного лечения метформин был назначен 99 пациентам (группа «М+»), суточная доза препарата составила 2000 [ 1000; 2000] мг. Из них в 31 случае метформин назначался впервые, при этом начальная доза препарата составляла 500 мг 1–2 раза в сутки с рекомендациями по дальнейшей титрации дозы до терапевтической. Из 68 пациентов, у которых применение метформина было возобновлено, в 55 случаях использовалась доза, используемая до госпитализации, и в 13 случаях потребовалось увеличение дозы метформина с 850 и 1000 мг до 2000 мг в сутки. В 22 случаях применение метформина не было возобновлено. Таким образом, 62 пациента составили группу «М-». Сравнительная клиническая характеристика групп «М+» и «М-» представлена в таблице 1. В группе «М+» медиана времени инициации терапии метформином составила 5 [ 4; 7] сут от момента поступления. В стационаре в группе «М+» монотерапия метформином проводилась в 11 случаях, терапия комбинацией с иНГЛТ-2 — в 17 случаях, комбинацией с препаратами сульфонилмочевины (СМ) — в 30 случаях, многокомпонентной комбинацией (метформин+СМ+иНГЛТ-2) — в 22 случаях.

Гликемический контроль. Применение метформина сопровождалось более качественным гликемическим контролем во время стационарного лечения по поводу острого ИМ в группе «М+» по сравнению с группой «М-»: достоверно более низкими уровнями средней гликемии, ее вариабельности, а также увеличением времени пребывания в целевом диапазоне гликемии во время стационарного лечения, результаты представлены в таблице 2. В группе «М+» TIR >55% достигли 61 (62%) пациент против 28 (45%) больных в группе «М-» (р=0,042, χ2 Пирсона). Количество случаев гипогликемии в группах достоверно не отличалось, из 16 случаев гипогликемии в 15 применялся инсулин и в 1 случае препарат сульфонилмочевины.

Кислотно-щелочное состояние. В группе «М+» на 3-и сутки от начала использования метформина отмечаются достоверные, но клинически незначимые изменения: 1) снижение рН c 7,40 [ 7,34; 7,42] при поступлении до 7,37 [ 7,36; 7,40] (p=0,009, критерий Вилкоксона) и 2) нарастание дефицита оснований BE с -0,8 [-1,9; 0,8] ммоль/л при поступлении до -1,3 [-2,4; -0,2] (p=0,011, Wilcoxon). При этом уровень лактата не увеличивался 1,4 [ 1,1; 1,7] vs 1,4 [ 1,1; 1,6] ммоль/л (p=0,799, критерий Вилкоксона). Случаев лактат-ацидоза в исследуемой когорте не выявлено.

Функция почек. Из 89 пациентов, использующих метформин до госпитализации, ОПП зарегистрировано в 14 случаях (16%); из 69 больных, не применявших метформин до поступления в стационар, ОПП развилось в 13 случаях (19%), p=0,692 (χ2 Пирсона). Таким образом, применение метформина до госпитализации по поводу ИМ (и, соответственно, непосредственно до проведения селективной коронарографии с использованием рентгеноконтрастных препаратов) не сопровождалось увеличением риска развития ОПП: ОР 0,85 (0,37–1,96), p=0,691. На фоне применения метформина в группе «М+» не выявлено достоверной динамики уровня креатинина от момента поступления к моменту выписки из стационара: 81,8 [ 72,9; 102,5] vs 94,6 [ 85,8; 117,1] мкмоль/л (р=0,404, критерий Вилкоксона).

Прогноз. В течение 12 мес наблюдения в исследуемой когорте зафиксировано 23 летальных исхода, из них 7 (7%) в группе «М+» и 16 (26%) в группе «М-», р<0,001 (χ2 Пирсона), рис. 1, А. Следует отметить, что все случаи смерти в стационаре (11 пациентов, летальность в общей когорте 7%) произошли в группе «М-». При анализе прогноза выписанных пациентов видно, что кривые выживаемости в группах «М+» и «М-» достоверно не расходятся (рис. 1, Б).

**Table:** Таблица 1. Сравнительная клиническая характеристика групп «М+» и «М-»

Параметр	«М+» (n=99)	«М-» (n=62)	р
Возраст, годы	64±9	68±9	0,005*
Мужчин /женщины, n (%)	49/50(49)/(51)	24/38(39)/(61)	0,181**
Срок госпитализации от момента начала симптомов, n (%)	<2 ч	15 (15)	4 (6)	0,193**
2–12 ч	60 (61)	33 (54)
12–24 ч	15 (15)	15 (24)
>24 ч	9 (9)	10 (16)
Инфаркт-связанная артерия, n (%)	ПНА	40 (40)	31 (50 %)	0,699**
ОА	14 (14)	6 (10)
ПКА	42 (43)	22 (35)
Острая сердечная недостаточность	ОЛЖН	4 (4)	12 (19)	0,002**
Кардиогенный шок	2 (2)	9 (15)	0,002**
Фибрилляция предсердий, n (%)	11 (11)	5 (8)	0,532**
Предшествующий ИМ в анамнезе, n (%)	13 (13)	13 (21)	0,193**
Длительность СД2, годы	7 [ 1; 10]	7 [ 0,1; 16]	0,505***
Впервые выявленный СД2, n (%)	16 (16)	9 (15)	0,712**
HbA1c, %	8,2±1,8	7,9±1,7	0,424*
ИМТ, кг/м2	30,1 [ 27,4; 35]	31,2 [ 26,9; 35]	0,412***
Предшествующая сахароснижающая терапия, n (%)	Инсулин	19 (19)	14 (23)	0,510**
Метформин	67 (68)	22 (37)	<0,001**
Препараты СМ	40 (41)	27 (45)	0,294**
иДПП-4	11 (11)	3 (6)	0,576**
иНГЛТ-2	3 (3)	2 (4)	0,932**
Сахароснижающая терапия в течение госпитализации, n (%)	Инсулин	67 (68)	53 (85)	0,011
Метформин	99 (100)	0	
Препараты СМ	52 (53)	25 (40)	0,134
иНГЛТ-2	39 (39)	12 (19)	0,008
ФВ, %	46,9±7,7	43,7±8,3	0,013*
Уровень креатинина при поступлении, мкмоль/л	90,5±26,3	109,9±59,6	0,007*
рСКФ при поступлении, мл/мин	72,4±20,4	64,1±27,5	0,035*
Уровень креатинина через 48 ч после ангиографии, мкмоль/л	93,2±21,3	122,9±64,9	<0,001*
рСКФ через 48 ч после ангиографии, мл/мин	68,7±17,9	57,7±25,8	0,002*
рН	7,40 [ 7,38; 7,42]	7,38 [ 7,34; 7,39]	0,039***
Лактат, ммоль/л	1,4 [ 1,1; 1,7]	1,5 [ 1,3; 2,5]	0,069***
BE, ммоль/л	-0,8 [-1,9; 0,8]	-2,2 [-3,3; 0,9]	0,125***
Максимальное значение тропонина I, пг/мл	29 926 [ 12 456; 50 000]	45 237 [ 20 998; 50 000]	0,063***
NT-proBNP, пг/мл	331 [ 92; 724]	551 [ 176; 1026]	0,067***
АЛТ, Ед/л	28,3 [ 21,2; 41,3]	27,3 [ 20,5; 44]	0,723***

**Table:** Таблица 2. Параметры гликемического контроля за время стационарного лечения по поводу острого ИМ в группах «M+» и «М-»

Параметр	«M+» (n=99)	«M-» (n=62)	p
Количество измерений гликемии на 1 пациента за время стационарного лечения	22 [ 15; 29]	18 [ 13; 29]	0,417***
Первое значение гликемии при поступлении, ммоль/л	13,8±4,9	13,2±5,3	0,455*
Средняя гликемия во время госпитализации, ммоль/л	9,3±1,6	10,3±2,3	0,002*
Вариабельность гликемии в течение госпитализации (SD), ммоль/л	2,87±1,06	3,26±1,8	0,049*
Доля измерений гликемии в диапазоне 6,1–10,0 ммоль/л во время госпитализации («hTIR»), %	60±18	48±23	<0,001*
Количество пациентов, имеющих хотя бы 1 измерение гликемии <3,9 ммоль/л, n (%)	9 (9)	7 (11)	0,603**

**Figure:**
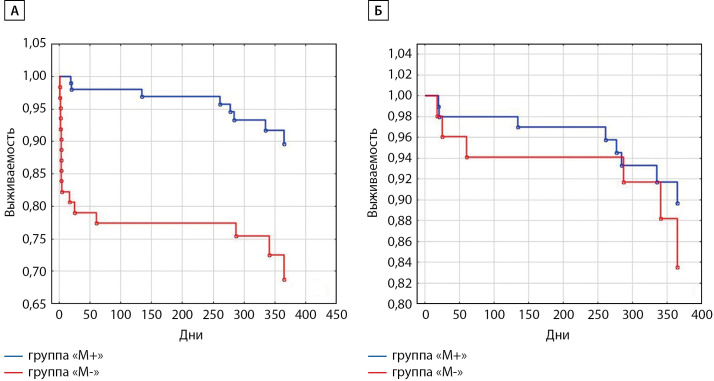
Рисунок 1. Кривые выживаемости (Kaplan-Meier) в течение года после госпитализации в группах «M+» и «М-». А — с учетом умерших в стационаре (р<0,001, Gehan’s Wilcoxon test); Б — только выписанных пациентов (р=0,490, Gehan’s Wilcoxon test).

## ОБСУЖДЕНИЕ

Клиническая значимость результатов

В исследовании получены важные с клинической точки зрения результаты. Во-первых, подтверждена безопасность проведения ангиографии на фоне непрерванного применения метформина. Рекомендация отменять метформин за 48 ч до планового введения рентгеноконтрастных препаратов основана на опасении потенцирования и повышения риска развития контраст-индуцированной нефропатии [17]. При остром ИМ с подъемом сегмента ST заранее прекратить прием метформина перед ангиографией по очевидным причинам невозможно. Проведенное исследование продемонстрировало, что применение метформина до госпитализации не сопровождалось увеличением риска развития ОПП после проведения ангиографии: ОР 0,85 (0,37–1,96), p=0,691. Полученный результат согласуется с недавними публикациями других авторов [18]. Во-вторых, применение метформина за пределами первых 48 ч после ЧКВ не сопровождалось повышением уровня лактата и клинически значимыми изменениями показателей pH и BE. Случаев лактат-ацидоза, равно как и предпосылок к его развитию, не выявлено. В-третьих, применение метформина закономерно сопровождалось более благоприятным профилем параметров гликемического контроля во время стационарного лечения (табл. 2) при условии, что исходно группы «М+» и «М-» качественно не отличаются по исходным параметрам СД2, таким как длительность диабета, уровень гликированного гемоглобина (HbA1c), предшествующая госпитализации терапия СД2 (табл. 1), уровень гликемии при поступлении (табл. 2). И, в-четвертых, применение метформина ассоциировалось с низкой частотой смертельных исходов в течение 12 мес (рис. 1, А), что перекликается с данными регистрового исследования, показавшего, что применение метформина снижает риск смерти от любых причин у пациентов с ОКС: ОР 0,50, 95% ДИ (0,26–0,95), p=0,035 [19]. И хотя в нашем исследовании такой результат получен в первую очередь за счет более тяжелого течения ИМ в группе «М-» и, как следствие, более высокой частоты летальных исходов во время госпитализации в этой группе, важно, что даже без учета случаев смерти в стационаре раннее назначение метформина при ИМ не сопровождалось ухудшением прогноза пациентов в течение 12 мес (рисунок 1, Б). Таким образом, проведенное исследование показывает безопасность применения метформина у пациентов с СД2 во время госпитализации по поводу ИМ.

Ограничения исследования

Рассматривать полученные результаты следует с учетом особенностей формирования групп. Метформин назначался пациентам по клиническим показаниям при отсутствии острой сердечной недостаточности, острых и хронических нарушений функции печени и почек, ограничивающих применение метформина согласно инструкции к препарату, при отсутствии кетоза и нормальном уровне лактата. Другими словами, распределение больных в группы «М+» и «М-» проводилось не случайным образом, а на основании клинической ситуации, что предопределило различия в группах (табл. 1). Можно заметить, что пациенты в группе «М-» характеризовались более тяжелым течением ИМ, более низким уровнем рСКФ исходно и более частым развитием ОПП к 3-м суткам от момента проведения ангиографии. Это объясняет более частое назначение инсулинотерапии в стационаре в группе «М-» (табл. 1). Обратим внимание, что среди пациентов, получавших метформин до госпитализации, у 22 больным прием препарата не был возобновлен во время стационарного лечения. Причинами этого служили осложненное течение острого ИМ (7 пациентов), сопутствующая острая госпитальная инфекция COVID-19 (5 пациентов), низкий уровень рСКФ (2 пациента), превышение уровня АЛТ более 3 референсных значений (2 пациента), наличие повышенного лактата в анализе КЩС и длительно сохраняющегося кетоза в моче (5 пациентов). Отсутствие рандомизации при формировании групп является важным ограничением проведенного исследования.

## ЗАКЛЮЧЕНИЕ

У больных СД2, госпитализированных по поводу ИМ, использование метформина ассоциировано с более качественным гликемическим контролем — достоверно более низкими уровнями средней гликемии, ее вариабельности, а также увеличением времени пребывания в целевом диапазоне гликемии во время стационарного лечения (hTIR). Проведение ангиографии у больных, исходно получающих регулярную терапию метформином, не сопровождается повышением риска развития ОПП. Назначение метформина на 3–7-е сутки после ангиографии не сопровождается повышением лактата и клинически значимыми отклонениями показателей КЩС. Применение метформина не приводит к ухудшению как краткосрочного, так и долговременного прогноза в течение 12 мес.

## ДОПОЛНИТЕЛЬНАЯ ИНФОРМАЦИЯ

Источники финансирования. Работа выполнена в соответствии с планом научной работы ФГБОУ ВО «ПИМУ» Минздрава России без привлечения иных источников финансирования.

Конфликт интересов. Авторы декларируют отсутствие явных и потенциальных конфликтов интересов, связанных с содержанием настоящей статьи.

Участие авторов. Коротина М.А. — сбор и обработка научного материала, написание текста и подготовка иллюстраций; Починка И.Г. — разработка концепции и дизайна исследования, редактирование текста; Стронгин Л.Г. — разработка концепции и дизайна исследования, редактирование текста. Все авторы одобрили финальную версию статьи перед публикацией, выразили согласие нести ответственность за все аспекты работы, подразумевающую надлежащее изучение и решение вопросов, связанных с точностью или добросовестностью любой части работы.
